# Intestinal Fistula: A Continuing Challenge: A Report of the Marjorie Budd Lecture

**Published:** 1988-11

**Authors:** Miles Irving, M.G. Wilson


### Intestinal Fistula: A Continuing Challenge

The 8th Marjorie Budd Lecture delivered on February 26th 1988 at the Bristol Royal Infirmary by **Professor Miles Irving** of Manchester.

Intestinal fistulae still present surgeons with a challenge caused by a combination of surgical, metabolic and ethical problems. In his opinion today the intellectual challenges of surgery principally lie outside the operating theatre and thosechallenges are becoming even greater as scientific knowledge advances. Today we must face the fact that what is theoretically feasible is not always practical or affordable.

The first book on intestinal fistulae was written by Thomas Pridgin Teale of Leeds in 1841. In his day most fistulae were the result of the natural progression of advanced disease. In the 1980s 70% of fistulae follow surgery and 20% are due to inflammatory bowel disease. The remaining 10% are the result of a variety of causes such as carcinoma. Thirty per cent of cases of Crohn's disease develop fistulae.

Fistulae may be external or internal. 70% of external fistulae will close spontaneously if correctly treated. However, internal fistulae virtually never do so and thus require operation should it be considered necessary to close them. The problems of external fistulation are many—a broken down abdominal wound after laparotomy, excoriation of the skin, metabolic, biochemical and nutritional disturbances and the underlying disease. The high output small bowel fistula is undoubtedly the most difficult to manage.

Eight years ago Professor Irving initiated a purpose built special unit for the investigation and treatment of 'intestinal failure'. The unit has four beds, a high nurse/patient ratio and all the necessary high technology. In 1980, the year of opening, the mortality of patients with intestinal failure for the preceding four years was 40%, during the next three years it was reduced to 21% and by 1986 to 7%.

His plans for the management of fistulae fell into four phases. The initial phase concentrated on restoring fluid volume, protecting the skin, determining electrolytes and assessing the extent of infection. This latter he considered especially important. He believed firmly that antibiotics should only be given for strictly specified indications and that their indiscriminate use led to dangerous superinfections, e.g. with fungi. Catheter infections were avoidable with strict nursing technique.

Phase two dealt with the correction of electrolyte imbalances, the treatment of sepsis, and the establishment of a feeding regimen. Whenever possible he advocated an oral liquid whole protein diet in preference to expensive proprietary preparations. When i-v feeding is needed it should not be given continuously but only at night, thus the patient's freedom of movement is not limited. He preferred a catheter inserted via the subclavian vein into the R atrium and tunnelled through the skin over the chest wall to emerge over the mid sternum. This should be used only for feeding and not for any other purposes such as the taking of blood samples or administration of drugs.

In the search for sepsis ultrasound and CT scans are of the greatest use and percutaneous drainage of intra-abdominal abscesses by radiologists has been 70% successful. Surgical drainage is only resorted to when this has failed.

Phase 3, from 48 hours to 5 days consists of the contribution of parenteral therapy and the establishment of the anatomy and the pathological basis of the fistula. All the previous phases should have been completed within 5 days. Phase 4 involves the maintenance of nutritional support andan assessment of the need for restorative surgery, resection or bypassing.

The concept of 'laparostomy' he considered of great value in patients with extensive persistent intra-abdominal sepsis. He illustrated it by describing a horrific case. In a young man, an oesophageal anastomosis for a benign condition had leaked. An inadvisable attempt to repair it had failed. An oesophagectomy and cervical oesophagostomy saved the patient's life but sequelae eventually included pancreatitis, gangrene of the stomach and of the transverse colon. After resection of the gangrenous viscera through a high transverse incision a duodenostomy was made and the wound was left open to granulate from the posterior abdominal wall. In 5 weeks it had healed. The patient was fed intravenously for a year and then successfully reconstructed by use of a colonic graft between the duodenum and the pharynx.

He recommended that anastomoses should not be made in severely malnourished or septic patients. Elective surgery today should be virtually complication-free, something that patients will come to demand. Anastomotic leakage should be avoidable, as long as we do not undertake anastomoses in the patient with hypoalbuminaemia. Hypoalbuminaemia is a marker of sepsis which is the principal determinant of success or failure. Inability to raise the serum albumin in response to treatment usually presages a fatal outcome.

In his own series a fistula patient with two organ/system failures and secondary haemorrhage never survived.

Practical tips included the use of the 'Parisian cocktail', for perfusing the gut and abscess cavities of patients with duodenal and gastric fistulae and associated peritonitis. He also commended the serosal patch method of closing fistulae which cannot be exteriorised and which sometimes succeeds when all else fails.

The formula for this "cocktail" described by Levy from Paris is:

| | |
|---|---|
| Aminocaproic acid | —4 g |
| Atropine sulphate | —0.25 mg |
| Thrombin | —5,000 units |
| Colloidal Silver | —100 mg |
| Isotonic Dialysate | —to 1,000 ml |

In a series of 100 cases of Crohn's disease, shortly to be reported, there was no mortality and there were no leaks. The fistula closure rate was 96%.

This talk, in spite of the philosophical preamble was intensely practical. Military stategists throughout history have proclaimed the same message, only attack when you have made every preparation and the ground is favourable, and sometimes the defensive build-up must be prolonged. Surgery guided by these principles will be that much safer.

M.G. Wilson.

